# A Small-Molecule Inhibitor of *T. gondii* Motility Induces the Posttranslational Modification of Myosin Light Chain-1 and Inhibits Myosin Motor Activity

**DOI:** 10.1371/journal.ppat.1000720

**Published:** 2010-01-15

**Authors:** Aoife T. Heaslip, Jacqueline M. Leung, Kimberly L. Carey, Federica Catti, David M. Warshaw, Nicholas J. Westwood, Bryan A. Ballif, Gary E. Ward

**Affiliations:** 1 Department of Microbiology and Molecular Genetics, University of Vermont, Burlington, Vermont, United States of America; 2 School of Chemistry and Centre for Biomolecular Sciences, University of St Andrews, North Haugh, St Andrews, Fife, Scotland, United Kingdom; 3 Department of Molecular Physiology and Biophysics, University of Vermont, Burlington, Vermont, United States of America; 4 Department of Biology and Vermont Genetics Network Proteomics Facility, University of Vermont, Burlington, Vermont, United States of America; Washington University School of Medicine, United States of America

## Abstract

*Toxoplasma gondii* is an obligate intracellular parasite that enters cells by a process of active penetration. Host cell penetration and parasite motility are driven by a myosin motor complex consisting of four known proteins: TgMyoA, an unconventional Class XIV myosin; TgMLC1, a myosin light chain; and two membrane-associated proteins, TgGAP45 and TgGAP50. Little is known about how the activity of the myosin motor complex is regulated. Here, we show that treatment of parasites with a recently identified small-molecule inhibitor of invasion and motility results in a rapid and irreversible change in the electrophoretic mobility of TgMLC1. While the precise nature of the TgMLC1 modification has not yet been established, it was mapped to the peptide Val46-Arg59. To determine if the TgMLC1 modification is responsible for the motility defect observed in parasites after compound treatment, the activity of myosin motor complexes from control and compound-treated parasites was compared in an *in vitro* motility assay. TgMyoA motor complexes containing the modified TgMLC1 showed significantly decreased motor activity compared to control complexes. This change in motor activity likely accounts for the motility defects seen in the parasites after compound treatment and provides the first evidence, in any species, that the mechanical activity of Class XIV myosins can be modulated by posttranslational modifications to their associated light chains.

## Author Summary

*Toxoplasma gondii* and related parasites within the Phylum Apicomplexa are collectively responsible for a great deal of human disease and death worldwide. The ability of apicomplexan parasites to invade cells of their hosts, disseminate through tissues and cause disease depends critically on parasite motility. Motility is driven by a complex of proteins that is well conserved within the phylum; however, very little is known about how the unconventional myosin motor protein at the heart of this motility machinery is regulated. *T. gondii* serves as a powerful model system for studying apicomplexan motile mechanisms. We show here that a recently identified pharmacological inhibitor of *T. gondii* motility induces a posttranslational modification of TgMLC1, a protein that binds to the myosin motor protein, TgMyoA. The compound-induced modification of TgMLC1 is associated with a decrease in TgMyoA mechanical activity. These data provide the first glimpse into how TgMyoA is regulated and how a change in the activity of the *T. gondii* myosin motor complex can affect the motility and infectivity of this important human pathogen.

## Introduction

*Toxoplasma gondii* is a protozoan parasite of the Phylum Apicomplexa. This phylum contains over 5,000 species, many of which are of significant medical or veterinary importance, including *Plasmodium spp.*, the causative agents of malaria, *Cryptosporidium spp.*, the causative agents of the diarrheal disease cryptosporidiosis, and *Eimeria spp.*, which cause losses in the US poultry industry exceeding $600 million annually.

Like most apicomplexans, *T. gondii* is an obligate intracellular parasite. Host cell invasion is a parasite-driven, multistep process that is necessary for parasite survival (reviewed in [1]). Prior to invasion, parasites glide along the surface of the host cell to be invaded, extending and retracting a tubulin-based cytoskeletal structure, the conoid, at their extreme apical tip [2]. Invasion is initiated by proteins released onto the parasite surface from apical secretory organelles known as the micronemes; these proteins mediate intimate and irreversible attachment to the host cell [3],[4]. At least one microneme protein also interacts with proteins secreted by a second set of apical organelles, the rhoptries, to form a ring-shaped zone of tight contact between the host cell plasma membrane (PM) and the PM of the internalizing parasite [5],[6]. As the parasite penetrates through this junction and into the host cell, it becomes enveloped by a parasitophorous vacuole membrane (PVM) that is derived primarily from the host cell PM [7]. In the final step of invasion, the PVM pinches off from the host cell PM to surround the fully internalized parasite.

Both gliding motility and host cell penetration are driven by the same unconventional Class XIV myosin motor protein, TgMyoA [8]. TgMyoA is a 93kDa protein consisting of a head domain, which contains only 23–34% identity to other myosin heavy chains, and a short neck/tail domain [9]. Although TgMyoA lacks a number of generally well conserved sequence features, such as a pair of cysteine residues in the converter domain and a glycine residue that acts as the “pivot-point” for the lever arm in most other myosin heavy chains [10],[11], it has a step size of 5.3nm and moves towards the plus-end of actin filaments at approximately 5 µm/s, a velocity comparable to skeletal muscle myosin [10].

**Figure 1:**
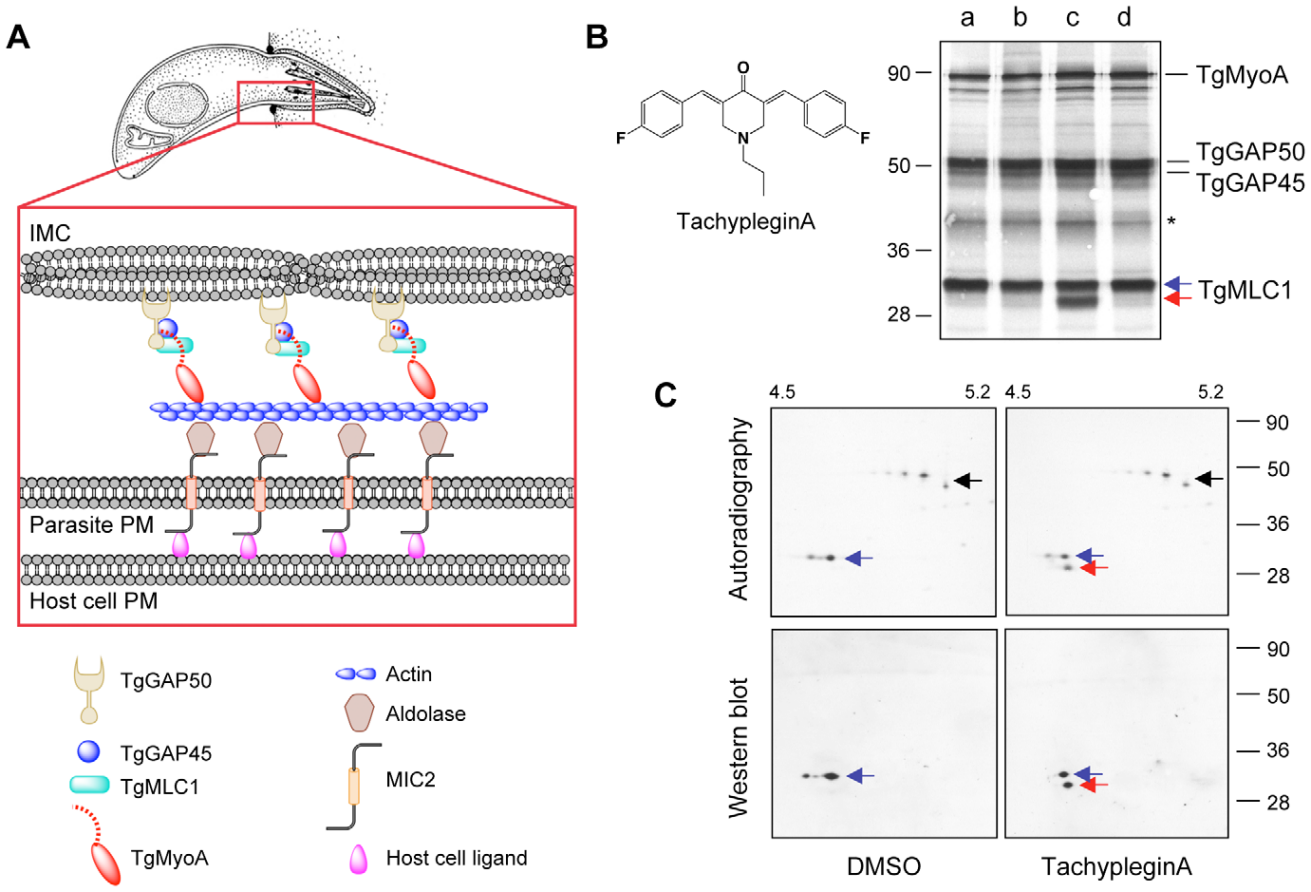
Treatment of parasites with tachypleginA induces a shift in the electrophoretic mobility of TgMLC1. **(A)** Current model for how the *T. gondii* myosin motor complex drives parasite motility. TgMyoA, TgMLC1 and TgGAP45 are anchored to the IMC via transmembrane protein TgGAP50. Short actin filaments located between the parasite plasma membrane and the IMC are connected to ligands on the host cell surface through bridging proteins TgMIC2 and aldolase. See text for further details. Adapted from [49]. **(B)** ^35^S-labeled extracellular parasites were treated for 15 minutes in medium containing: (a) DMSO; (b) 100 µM Inhibitor 22 (ref [26]); (c) 100 µM tachypleginA (structure shown in inset); or (d) 100 µM Enhancer 5 (ref [26]). Myosin motor complexes were isolated from the treated parasites by anti-TgGAP45 immunoprecipitation, and the immunoprecipitated proteins were resolved and visualized by SDS-PAGE / autoradiography. Treatment with tachypleginA results in an extra 30kDa protein associating with the myosin motor complex (red arrow). Numbers on the left indicate molecular mass in kDa. Asterisk indicates a degradation product of TgGAP45 [14]. **(C)** Myosin motor complexes were isolated from DMSO- and tachypleginA-treated, ^35^S-labeled extracellular parasites by anti-TgGAP45 immunoprecipitation. Proteins were resolved by 2D gel electrophoresis and visualized by autoradiography (upper panel) followed by western blotting with anti-TgMLC1 (lower panel). The 30kDa protein that appears after compound treatment is recognized by the anti-TgMLC1 antibody (red arrow), indicating that a modified form of TgMLC1 associates with the myosin motor complex after tachypleginA treatment. Unmodified TgMLC1 is indicated with a blue arrow and TgGAP45 is indicated with a black arrow. Although the phosphorylated form of TgMLC1 was below the limit of detection by western blotting after tachypleginA treatment in this particular experiment, this was not usually the case (*e.g.,* see Fig. S2). Numbers on the top indicate the pH gradient used for isoelectric focusing, and numbers on the right indicate molecular mass in kDa.

The short neck/tail domain of TgMyoA binds a single, calmodulin-like myosin light chain, TgMLC1 [10]. These two proteins associate with two additional proteins, TgGAP45 and TgGAP50, to form the myosin motor complex ([12],[13]; see Fig. 1A). TgGAP45 contains predicted palmitoylation and myristoylation sites and functions in motor complex assembly [14],[15], while TgGAP50 is a transmembrane protein that is thought to anchor the motor complex into the inner membrane complex (IMC) (Fig. 1A; [13],[14]). The motor complex is firmly immobilized in the IMC within cholesterol-enriched microdomains [16]. Short actin filaments located between the parasite PM and the IMC are connected to ligands on the host cell surface through a number of bridging proteins, including TgMIC2 and aldolase ([17]; Fig. 1A); these proteins, together with the myosin motor complex, are collectively referred to as the glideosome [12],[13]. During invasion, when TgMyoA anchored into the IMC undergoes its power stroke, the parasite is driven through the ring-shaped junction and into the host cell.

The components of the myosin motor complex are highly conserved across apicomplexan parasites [13],[18], and myosin-based motility is essential not only for invasion but also for penetrating biological barriers and disseminating through tissues during infection [8],[18],[19]. While the components of the motor complex have been well characterized, nothing is currently known about how the activity of the complex is regulated to generate the different speeds and types of motility that the parasite is capable of [20]. Myosin regulation in other systems can occur through heavy chain phosphorylation, which can alter the actin-activated ATPase activity of the myosin, its localization in the cell or its assembly with other myosin subunits (reviewed in [21],[22]). Myosin light chains also play a major role in regulating the ATPase activity and stability of myosin motor proteins [23]. The effect of a particular light chain on myosin function is regulated by calcium binding to the light chain and/or phosphorylation of the light chain by myosin light chain kinases, whose activities are themselves regulated by intracellular calcium levels and a variety of other signaling pathways [24]. The myosin light chain of *P. falciparum* (named MTIP, for myosinA tail domain-interacting protein) was recently shown to be phosphorylated [25], but whether or how the myosin light chains of apicomplexan parasites contribute to the regulation of Class XIV myosin motor function is unknown.

In a recent high-throughput screen, we identified 24 novel small-molecule inhibitors and six enhancers of *T. gondii* invasion [26]. Surprisingly, 21 out of the 24 invasion inhibitors inhibited parasite motility and all six enhancers of invasion enhanced parasite motility. This led us to hypothesize that some of these small molecules exert their effects by altering the composition or function of the *T. gondii* myosin motor complex. We show here that treatment of parasites with one of the motility inhibitors results in a posttranslational modification to TgMLC1. Furthermore, we show that motor complexes containing the altered form of TgMLC1 have reduced mechanical activity in an *in vitro* motility assay. This change in TgMyoA motor activity likely accounts for the motility defects seen in the parasite after compound treatment and provides the first evidence, in any apicomplexan parasite, for the modulation of Class XIV myosin activity by myosin light chain(s).

## Results

### A small-molecule inhibitor of *T. gondii* invasion and motility alters the electrophoretic mobility of TgMLC1

To test whether any of the inhibitors or enhancers of parasite motility identified in our screen affect the composition of the myosin motor complex, ^35^S-labeled extracellular parasites were treated with each of the small molecules and the proteins of the motor complex were isolated by co-immunoprecipitation with an anti-TgGAP45 antibody, resolved by SDS-PAGE and visualized by autoradiography. In all cases, the four major components of the motor complex (TgMyoA, TgGAP50, TgGAP45 and TgMLC1) were recovered (Fig. 1B). While 26 of the 27 compounds tested had no apparent effect on motor complex composition, treatment with one of the motility inhibitors (referred to previously as Inhibitor 24 [26]), resulted in the association of a prominent new 30kDa protein with the motor complex (Fig. 1B, red arrow). We have named this motility inhibitor tachypleginA (“tachy” for tachyzoite, “plegin” from the Greek-derived suffix “plegia”, for paralysis).

The motor complex-associated proteins were then resolved by 2D gel electrophoresis. TgGAP45, which is multiply phosphorylated [15], resolves as a prominent charge train (Fig. 1C, upper panels, black arrow). TgMLC1 from DMSO-treated parasites runs as two distinct spots (Fig. 1C, blue arrow), the more minor and acidic of which is phosphorylated (ATH, BAB and GEW, unpublished data). After treatment with tachypleginA, a new ^35^S-labeled spot is apparent immediately under the TgMLC1 spots (Fig. 1C, upper panels, red arrow). Western blotting with an antibody against TgMLC1 identified this new motor complex-associated protein as a modified form of TgMLC1 (Fig. 1C, lower panels), a result subsequently confirmed by mass spectrometry (see below).

**Figure 2:**
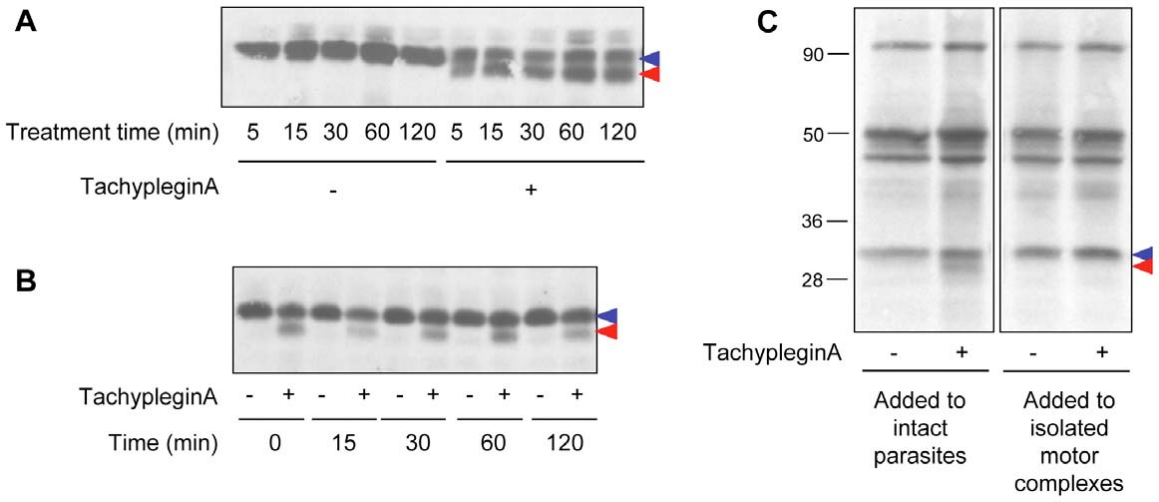
The effect of tachypleginA on TgMLC1 is rapid, irreversible and only occurs in intact parasites. **(A)** Extracellular parasites were treated with 100 µM tachypleginA or an equivalent amount of DMSO for times ranging from 5 to 120 minutes. Parasite proteins were resolved by SDS-PAGE and TgMLC1 was visualized by anti-TgMLC1 western blot. The unmodified and modified forms of TgMLC1 are indicated with blue and red arrows, respectively. **(B)** Intracellular parasites were treated with 100 µM tachypleginA or an equivalent amount of DMSO for 15 minutes. The compound was removed and parasites were incubated at 37°C in compound-free media for the times indicated. Parasite proteins were resolved by SDS-PAGE and visualized by anti-TgMLC1 western blot. The unmodified and modified forms of TgMLC1 are indicated with blue and red arrows, respectively. **(C)** ^35^S-labeled extracellular parasites were treated with 100 µM tachypleginA or an equivalent amount of DMSO for 20 minutes at 25°C and motor complexes were isolated by anti-TgGAP45 immunoprecipitation (left panel). Alternatively, motor complexes were isolated from ^35^S-labeled extracellular parasites and treated with DMSO or 100 µM tachypleginA for 20 minutes at 25°C (right panel). Proteins were resolved by SDS-PAGE and visualized by autoradiography. The unmodified and modified forms of TgMLC1 are indicated with blue and red arrows, respectively. Numbers on the left indicate molecular mass in kDa.

The TgMLC1 modification occurs within five minutes of compound treatment in both extracellular (Fig. 2A) and intracellular (data not shown) parasites. The modification does not reverse to any significant extent, even two to four hours after compound washout (Fig. 2B and data not shown). TachypleginA is only able to induce the mobility shift when added to intact parasites, not to parasite lysates or isolated motor complexes (Fig. 2C). Strikingly, only ∼50% of the TgMLC1 is modified in response to tachypleginA (Figs. 1B & C, 2A–C), even after multiple treatments and extended treatment times.

A collection of analogs of tachypleginA was generated using parallel synthesis techniques, and each analog was tested for its effect on invasion, motility and the TgMLC1 electrophoretic mobility shift. While the majority of the analogs tested did not score as inhibitors in the invasion assay (data not shown), we identified two analogs (tachypleginA-2 and tachypleginA-3; Fig. S1 and Text S1) that inhibited parasite invasion and motility and reproducibly induced the TgMLC1 mobility shift. It was difficult to establish structure-activity relationships among these analogs due to the semi-quantitative nature of the *in vivo* assays [27] and problems with compound stability and solubility. However, we did not identify any analogs that induced the modification of TgMLC1 without also inhibiting motility and invasion, consistent with the hypothesis that the observed block in these processes results from the modification of TgMLC1.

### Mapping the site of modification

**Figure 3:**
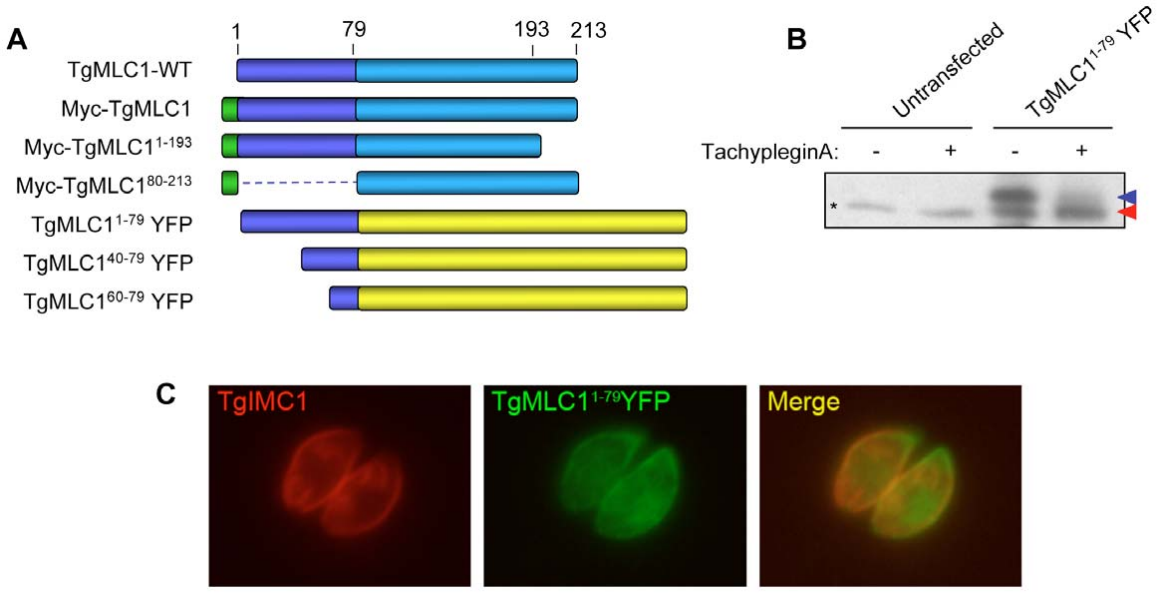
Amino acids 1–79 of TgMLC1 are sufficient for the tachypleginA-induced mobility shift. **(A)** Schematic of the TgMLC1 truncation mutants generated. **(B)** Parasites transiently expressing TgMLC1^1–79^YFP were treated with 100 µM tachypleginA or an equivalent amount of DMSO. Proteins were resolved by SDS-PAGE and visualized by anti-GFP western blot. The unmodified and modified forms of TgMLC1^1–79^YFP are indicated with blue and red arrows, respectively. Asterisk (*) indicates a protein that is non-specifically recognized by the anti-GFP antibody. **(C)** Dual label immunofluorescence of intracellular parasites expressing TgMLC1^1–79^YFP, using antibodies against TgIMC1 and GFP. TgMLC1^1–79^YFP largely colocalizes with TgIMC1, indicating that the first 79 amino acids of TgMLC1 are sufficient for peripheral localization.

An epitope-tagged copy of TgMLC1 (Myc-TgMLC1) was expressed in the parasites and shown to be modifiable by tachypleginA treatment (Fig. S2). As a first step towards identifying the site of modification, Myc-TgMLC1 truncation mutants were generated by deletion of 79 amino acids from the N-terminus or 20 amino acids from the C-terminus (Fig. 3A). Parasites stably expressing these truncations could not be generated, and insufficient protein was isolated from transiently expressing parasites for analysis of the electrophoretic mobility of the truncated proteins in response to tachypleginA. Immunofluorescence analysis of individual, transiently expressing parasites showed that while both full length TgMLC1 and Myc-TgMLC1^1–193^ localize to the parasite periphery, Myc-TgMLC1^80–213^ is distributed throughout the cytosol (Fig. S3), suggesting that amino acids 1–79 of TgMLC1 are necessary for proper localization.

Based on these preliminary truncation data, different N-terminal fragments of TgMLC1 were fused to yellow fluorescent protein (YFP) and the localization and ability of the fusion proteins to undergo an electrophoretic mobility shift in response to tachypleginA treatment were determined. A fusion protein containing amino acids 1–79 of TgMLC1 (TgMLC1^1–79^YFP) was found to largely colocalize with the IMC marker IMC1 (Fig. 3C), confirming that the N-terminal 79 amino acids are sufficient for TgMLC1 localization to the parasite periphery. Importantly, TgMLC1^1–79^YFP also undergoes an electrophoretic mobility shift in response to tachypleginA (Fig. 3B), suggesting that the site(s) of modification lie(s) within these first 79 amino acids. Further truncations within these first 79 amino acids were uninformative, as TgMLC1^40–79^YFP and TgMLC1^60–79^YFP localized to the cytosol, and neither underwent an electrophoretic mobility shift in response to tachypleginA treatment (data not shown).

Next, we used a quantitative mass spectrometry approach (Stable Isotope Labeling by Amino Acids in Culture, or SILAC [28]) to map the site of modification by searching for predicted tryptic peptides within the first 79 amino acids of both the upper and the lower, compound-induced forms of TgMLC1. Any peptide detected at its expected mass in one of the TgMLC1 forms but not the other could potentially contain the site of modification. Parasites were cultured for four days in either “heavy” medium (*i.e.*, medium containing ^13^C_6_-, ^15^N_4_-arginine and ^13^C_6_-, ^15^N_2_ -lysine) or “light” medium (containing only natural amino acids). A four-day culture labels parasite proteins in the heavy sample to near completion, but still leaves a small portion of the proteins unlabeled, which was important for quantitation (see below). The parasites cultured in heavy media were treated with tachypleginA and the parasites cultured in light media with an equivalent amount of DMSO. The myosin motor complexes from each population were then isolated and run together on the same 2D gel. The upper spot on such a 2D gel should contain a mixture of both heavy and light TgMLC1 while the lower spot should contain primarily heavy TgMLC1 (and smaller amounts of light TgMLC1, since the labeling was not driven to completion). The upper and lower spots were excised from the gel, digested with trypsin and subjected to liquid chromatography-tandem mass spectrometry (LC-MS/MS). Since heavy and light peptides of the same sequence have identical chromatographic profiles but different masses, the relative abundance of each peptide in its heavy and light forms can be readily determined. Owing to the natural abundance of ^13^C, each peptide is observed as a cluster of peaks with the light cluster spaced apart from the heavy cluster, consistent with the mass and number of heavy arginine and lysine residues found in each peptide.

**Figure 4:**
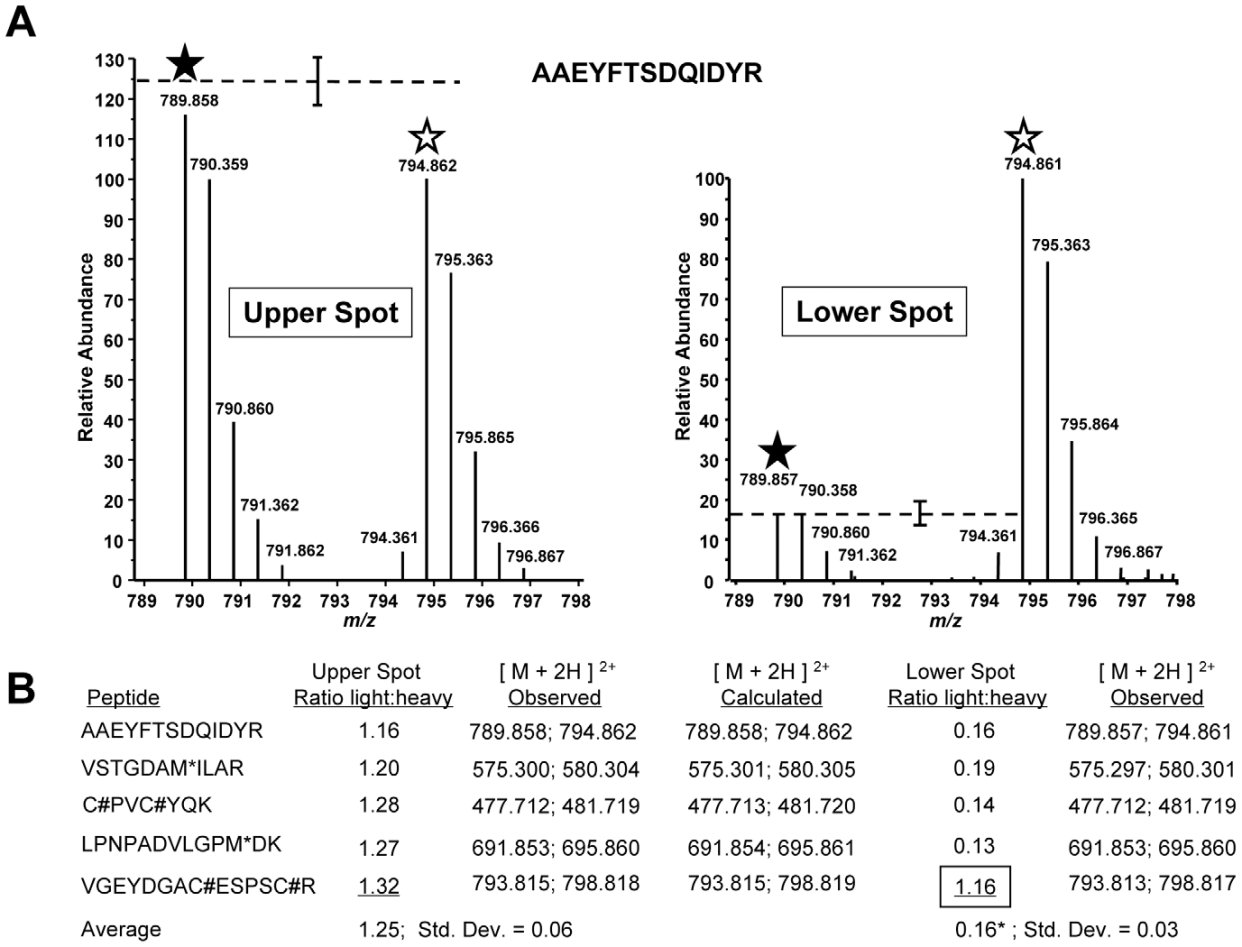
TachypleginA promotes the apparent posttranslational modification of TgMLC1 peptide V_46_GEYDGACESPSCR_59_. TgMLC1 from tachypleginA- and DMSO-treated parasites was isolated by immunoaffinity chromatography and 2D gel electrophoresis, and subject to LC-MS/MS (see text for details). **(A)** The averaged light and heavy isotopic envelopes for one peptide (A_190_AEYFTSDQIDYR_202_) from the upper TgMLC1 spot (left panel) and lower, tachypleginA-induced spot (right panel). SILAC ratios were generated by comparing the relative abundances of the monoisotopic light and heavy peaks (filled and open stars, respectively). The dashed lines indicate the average relative abundances of the light *vs.* heavy monoisotopic peaks of the peptides listed in Fig. 4B (average abundance of heavy peaks set to 100; error bars indicate the standard deviation from the mean). **(B)** The SILAC ratios (light:heavy) for the five most readily identifiable peptides in the upper and lower spots. The average SILAC light:heavy ratio for the upper spot (1.25) was calculated using the SILAC ratios of all five listed peptides, whereas the average for the lower spot (0.16*) was calculated omitting the data for peptide V_46_GEYDGACESPSCR_59_ as its SILAC ratio (1.16, boxed) suggested it was a contaminant from the upper spot. M* indicates an oxidized methionine residue. C# indicates a carbamidomethyl cysteine residue generated by alkylation with iodoacetamide prior to running the 2D gels. See Fig. S4 for the low energy CID MS/MS spectrum of V_46_GEYDGACESPSCR_59_ and Fig. S5 for a semi-quantitative measurement of the relative amount of this peptide present in the upper and lower spots.

Multiple TgMLC1 peptides were identified from each spot. The five most readily identified peptides in the upper spot revealed similar light to heavy ratios, with the light peptide averaging 1.25 times more than the heavy peptide (Fig. 4B). When the relative abundances of these same peptides were examined in the lower spot, four of the five showed a consistent but very different ratio from the peptides in the upper spot with an average light:heavy ratio of 0.16 (Fig. 4A,B). The fifth peptide (V_46_GEYDGACESPSCR_59_), however, showed the same ratio as that observed in the upper spot. Furthermore, uncharacteristic of the other four peptides, this fifth peptide was chosen by the instrument for fragmentation six times fewer in the lower spot compared to the upper spot, suggesting it was more abundant in the upper spot. This suggestion was further supported when the relative abundances of the peptide in the upper and lower spots were compared to the relative abundance of a polydimethylcyclosiloxane background ion (Si(CH_3_)_2_O)_6_^+^) that is commonly used as an internal calibrant for mass spectrometry [29]. Using this background ion as a reference, we estimated that the peptide V_46_GEYDGACESPSCR_59_ was approximately 225-fold more abundant in the upper form than in the lower form (Fig. S5). Together, these data provide strong evidence that the trace amount of this peptide detected in the lower form was in fact a contaminant from the upper form. Thus, while the unmodified form of the peptide V_46_GEYDGACESPSCR_59_ can be readily detected in the upper TgMLC1 spot, it is effectively undetectable in the lower compound-induced spot at its predicted (unmodified) mass and therefore likely contains the site of compound-induced modification. These data also suggest that compound treatment induces the modification of this peptide, rather than removal of a modification already present on the upper form of TgMLC1.

In an attempt to identify the precise site of modification, we conducted LC-MS/MS analysis on the lower, compound-induced spot of TgMLC1. Differential Sequest searches were performed for potential phosphorylation of the peptide V_46_GEYDGACESPSCR_59_ on serine, or tyrosine, as well as acetylation or mono-, di-, and tri-methylation of arginine. Only the unmodified peptide V_46_GEYDGACESPSCR_59_ (alkylated during the reaction with iodoacetamide prior to resolution by 2D gel electrophoresis) was identified, which, given the SILAC data described above, was presumably due to trace contamination from the upper spot. Given the many different posttranslational modifications that can occur on cysteine [30], the data were also subjected to a series of Sequest searches that permitted dynamic modification of Cys53 and/or Cys58 with mass values from 1 to 353 (the mass of tachypleginA). Again, only trace contamination of unmodified peptide was detected. Further analytical work will be required to determine the precise site and nature of the modification, but these data suggest either an uncommon posttranslational modification or one that renders the peptide difficult to analyze by LC-MS/MS.

### The effect of tachypleginA on myosin motor complex function

**Figure 5:**
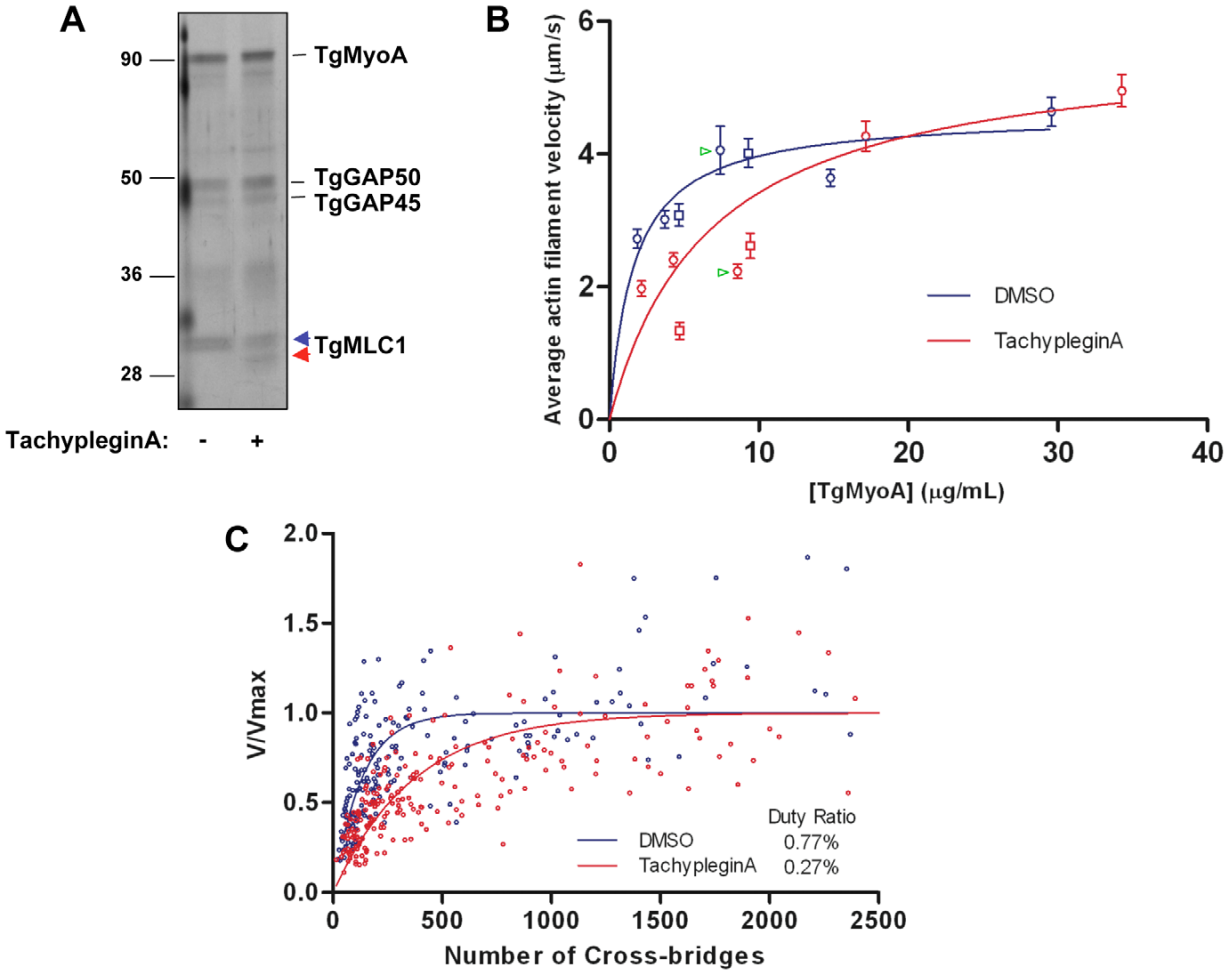
TachypleginA treatment causes a decrease in the motor activity of the TgMyoA motor complex. **(A)** Affinity-purified myosin motor complexes from DMSO- and tachypleginA-treated parasites, resolved by SDS-PAGE and visualized by silver stain. The unmodified and modified forms of TgMLC1 are indicated with blue and red arrowheads, respectively. Numbers on the left indicate molecular mass in kDa. **(B)** Mean actin filament displacement velocities at different TgMyoA concentrations. Red symbols indicate motor complexes isolated from tachypleginA-treated parasites; blue symbols indicate motor complexes isolated from DMSO-treated parasites. Squares and circles indicate data from two independent experiments, error bars denote standard error of the mean, and green arrowheads indicate samples shown in Supplemental Videos S1 and S2. Each data point from the DMSO-treated samples was compared to the corresponding data point from the tachypleginA-treated sample that was most similar in TgMyoA concentration, using an independent two-tailed t-test. At all low (<10 µg/ml) TgMyoA concentrations, motor complexes isolated from tachypleginA-treated parasites displaced actin filaments at a significantly slower velocity than motor complexes isolated from DMSO-treated parasites (p<0.001). In contrast, at higher TgMyoA concentrations (>15 µg/ml), no statistically significant differences in displacement velocity were observed. The data were fit by nonlinear regression to a simple hyperbola. **(C)** Actin filament velocity (V/V_max_) was plotted as a function of the number of myosin heads capable of interacting with the actin filament. The calculated duty ratios (see Methods) were significantly different for TgMyoA from untreated and tachypleginA-treated parasites (0.77±0.07% [best fit ± standard error; 95% confidence interval 6.3–9.1%] and 0.27±0.02% [95% confidence interval 2.3–3.1%], respectively).

To determine if the TgMLC1 modification affects TgMyoA activity, parasites stably expressing FLAG-tagged TgMLC1 (FLAG-TgMLC1-WT) were generated. Highly enriched myosin motor complexes could then be isolated from DMSO- and tachypleginA-treated parasites by anti-FLAG affinity chromatography (Fig. 5A). The concentration of TgMyoA in each preparation was determined by comparing the fluorescence intensity of the TgMyoA band to that of protein standards in SYPRO Ruby-stained gels (Fig. S6).

The motion-generating capacity of the myosin motor complex was measured in an *in vitro* motility assay, in which isolated motor complexes were adsorbed to the surface of a nitrocellulose-coated glass coverslip and the velocity with which these motor complexes moved fluorescently labeled chicken skeletal muscle actin filaments was determined (Videos S1 & S2; see also refs. [10],[31],[32]). When adsorbed at low densities to the coverslip (<10 µg/ml TgMyoA), complexes from tachypleginA-treated parasites were found to displace actin filaments with a significantly lower velocity than those from untreated control parasites (Fig. 5B). As the motor density increased, the difference in velocities became progressively smaller, and by 15 µg/ml TgMyoA the velocities of actin filament displacement by control and tachypleginA-treated motor complexes were statistically indistinguishable (Fig. 5B).

Maximal actin filament velocity (V_max_) in the motility assay is achieved when at least one myosin head is strongly bound to the actin filament and generating motion at every point in time [33]. The fraction of myosin's catalytic cycle that the motor is strongly bound to actin is defined as its duty ratio [34]. Therefore, myosins having low duty ratios (≤5%) require higher densities of myosin on the motility surface to achieve continuous actin filament movement at V_max_. The observation that, at low densities, TgMyoA from tachypleginA-treated parasites generates lower filament velocities than the same concentration of TgMyoA from control parasites suggests that the population of TgMyoA in the tachypleginA-treated samples has a lower duty ratio than TgMyoA from the DMSO-treated samples, thus requiring more TgMyoA to achieve V_max_.

The TgMyoA duty ratio was determined by calculating the number of myosin molecules on the motility surface that are available to interact with a given actin filament and plotting this number against the measured displacement velocity associated with that filament (see Materials and Methods; [33]). The data were fit to the equation: V =  (*a*×V_max_)×[1−(1−f)^n^], where f is the duty ratio, n is the number of myosin heads, and *a* is an efficiency factor for coupling myosin-generated motion to actin filament movement at low myosin densities [33],[35]. Based on these fits (Fig. 5C), TgMyoA is characterized by an extremely low duty ratio of 0.77±0.07% (best fit ± standard error). Treatment with tachypleginA decreases the duty ratio of the population by more than 60%, to 0.27±0.02%.

## Discussion

TachypleginA is a recently identified inhibitor of *T. gondii* motility and invasion [26]. We show here that treatment of parasites with tachypleginA results in a modification to TgMLC1 that alters its electrophoretic mobility. Early attempts to identify the nature of the modification ruled out changes in phosphorylation state, N- or C-terminal proteolysis and differential splicing (data not shown). Truncation analysis showed that the residue(s) responsible for the mobility shift lie(s) within the first 79 residues of the protein. Subsequent quantitative proteomic analysis identified V_46_GEYDGACESPSCR_59_ as the likely modified peptide. No common posttranslational modifications on specific residues within this peptide could be identified. We are continuing to pursue the nature of the modification but anticipate that it may either be a novel type of posttranslational modification, or that the modified peptide is selectively lost during sample preparation or does not ionize or fragment well for mass spectrometry analysis.

It is difficult to predict how a posttranslational modification within the peptide V_46_GEYDGACESPSCR_59_ of TgMLC1 could cause a change in TgMyoA motor activity. Immunoprecipitation experiments showed that the four main components of the glideosome remain associated after tachypleginA treatment (Fig. 1A and data not shown), indicating that drug treatment has no appreciable effect on motor complex stability. Furthermore, actin cosedimentation assays performed in the absence of ATP, *i.e.*, conditions under which myosin binds strongly to actin, show that all of the detectable TgMLC1 in the parasite pellets with actin after treatment with either DMSO or tachypleginA, indicating that the binding of TgMLC1 to TgMyoA is not disrupted by compound treatment (data not shown). TgMLC1 and its homologs in other apicomplexan parasites contain a 70–82 amino acid N-terminal extension (70 aa in TgMLC1) that is not found in most other calmodulin-like myosin light chains. While the crystal structure of *Plasmodium yoelii* MyoA_tail_ bound to *P. falciparum* myosin light chain (PfMTIP) lacking the majority of its N-terminal extension (amino acids 1–62) has been solved [36], the structures of full length PfMTIP and TgMLC1 have not. It is therefore not known how the N-terminal extensions of these light chains are oriented in relation to MyoA. Essential light chain from fast skeletal muscle (ELC1) contains a 40 amino acid lysine-rich N-terminal extension compared to ELC3, and this positively charged N-terminal extension interacts with the negatively charged C-terminus of actin [37]. TgMLC1 contains three lysine residues near its N-terminus, so it is possible that TgMLC1 modulates myosin motor complex activity through a similar direct interaction with actin. Experiments are currently underway to test this hypothesis; if correct, the tachypleginA-induced modification could disrupt this interaction.

It is not understood why only half of the TgMLC1 becomes modified after compound treatment. The ratio of TgMyoA:TgMLC1:TgGAP45:TgGAP50 in the myosin motor complex is thought to be 1∶1∶1∶1 [14]. However, if individual motor complexes were to stably associate with one another, the factor responsible for the modification might have access to some but not all of the TgMLC1 molecules. When Myc-tagged TgMLC1 was expressed in parasites (which also express untagged, endogenous TgMLC1), affinity purification of myosin motor complexes by anti-Myc immunoprecipitation brought down Myc-tagged, but not untagged TgMLC1 (data not shown), suggesting that individual motor complexes do not physically associate with each other. Alternatively, the compound might be compartmentalized within the parasite or the efficiency with which TgMLC1 becomes modified could be influenced by other posttranslational modifications. The phosphorylated form of TgMLC1 does not shift downward in response to tachypleginA (*e.g.*, Figs. 1C & S2), suggesting that the unphosphorylated form of TgMLC1 is indeed preferentially modified. Further work will be required to identify all the posttranslational modifications present on TgMLC1 and to determine whether some combination of pre-existing modifications is responsible for the observation that the stoichiometry of modification never reaches 100%. It is intriguing to note that when amino acids 1–79 of TgMLC1 were fused to YFP, the electrophoretic mobility shift occurred to near completion in the presence of compound (Fig. 3B). This could indicate that the C-terminus of the protein serves an autoinhibitory function, blocking the modification; the extent of the autoinhibition could depend on other posttranslational modifications.

Regardless of the reason for the substoichiometric modification of TgMLC1, given that the myosin motor complexes isolated from tachypleginA-treated parasites and analyzed in the *in vitro* motility assays contained a roughly 1∶1 ratio of modified:unmodified TgMLC1, it is likely that the modification has an even more profound effect on TgMyoA function than described here. Indeed, the decrease in calculated duty ratio for the ensemble of myosin motor complexes after tachypleginA treatment could reflect the mechanical capacity of a heterogeneous population of motor complexes, half of which behave like wild-type complexes while the other half (with a modified TgMLC1) do not contribute substantially to actin filament movement. This simple scenario could account for the apparent reduction in duty ratio by effectively reducing the number of active motors on the motility surface.

TachypleginA does not alter the electrophoretic mobility of TgMLC1 when added to cell extracts or isolated myosin motor complexes, nor does it affect TgMyoA activity when added directly to the *in vitro* motility assay. It is possible that tachypleginA or a metabolic product of tachypleginA binds directly to TgMLC1 in intact parasites to cause the electrophoretic mobility shift, and that some parasite factor required for this to occur is disrupted or lost during parasite extraction. Alternatively, TgMLC1 may not be the direct target of the compound, but rather becomes modified in intact parasites as a downstream consequence of tachypleginA interacting with some other target(s) within the parasite. In support of this hypothesis, we have shown that tachypleginA also inhibits microneme secretion [26]. Since microneme secretion does not require a functional myosin motor complex [8]), this observation suggests that tachypleginA perturbs more than one signaling pathway within the parasite. Disruption of these other pathways may contribute to the inhibition of invasion and motility in the presence of tachypleginA. Attempts to identify the direct target of tachypleginA are currently underway, and are likely to provide new insights into the signaling pathways that control parasite motility and microneme secretion.

Studies of myosin motor complex function and regulation are important for two reasons. From a biophysical viewpoint, many questions remain about how the Class XIV myosins, which have a number of unusual features compared to other myosins [9],[10], generate the force required to propel the parasites during gliding motility and invasion. Secondly, because Class XIV myosins are found in apicomplexans and their close phylogenetic relatives, but not in humans (see [38] for review), and are essential for parasite survival [8], they represent potential new drug targets. We have shown here that the machinery that regulates TgMyoA function is susceptible to pharmacological perturbation (see also [39]). A better understanding of how Class XIV myosins function and how they are regulated by their associated light chains may lead to new chemotherapeutic approaches to the treatment of disease caused by *T. gondii* and other apicomplexan parasites.

## Materials and Methods

### Cell culture

Parasites were maintained by continuous passage in human foreskin fibroblasts (HFFs) in Dulbecco's Modified Eagle's Media (DMEM) containing 1% (v/v) heat inactivated fetal bovine serum (FBS), 1% (v/v) penicillin/streptomycin mix (Invitrogen, Carlsbad CA) and 1% (v/v) 10 mM HEPES pH 7.0 (Invitrogen) as previously described [40].

### Compound storage and use

All small-molecule inhibitors were either purchased from ChemBridge Corporation (San Diego CA) or synthesized as described and stored at −20°C. Compound stock solutions were dissolved to a concentration of 40 mM in high quality DMSO and stored in the dark at −20°C. Unless otherwise noted, compounds were diluted to a concentration of 100 µM in Hanks Balanced Salt Solution immediately before use. Invasion and motility assays were performed as previously described [26].

### Western blotting

Protein samples were boiled in 1X SDS-PAGE sample buffer in the presence of 1.25% (v/v) β-mercaptoethanol for five minutes. Proteins were either visualized directly by staining with silver [41], Colloidal Coomassie Blue or SYPRO Ruby (Bio-Rad, Hercules CA), or transferred to nitrocellulose or PVDF [42]. For western blot analysis, membranes were blocked with 5% (w/v) dry milk in Tris-buffered saline containing 0.1% (v/v) Tween (TBS-T) for one hour to overnight. Blots were incubated with primary antibody for one hour and secondary antibody conjugated to horseradish peroxidase for 30 minutes, agitating at 25°C. Anti-TgMLC1 antibody was diluted 1∶2,000. Anti-GFP (Invitrogen) and anti-Myc monoclonal antibody 9E10 (Developmental Studies Hybridoma Bank, Iowa City IA) were diluted 1∶1,000.

### 2D gel electrophoresis

Protein pellets were resuspended in lysis buffer (10 mM Tris pH 7.4 containing 0.5% (v/v) protease inhibitor mix (Sigma Catalog # P8340, St. Louis MO) and 50 µg/ml RNase/DNase), followed by four freeze/thaw cycles in liquid nitrogen. SDS buffer (0.3% (w/v) SDS and 200 mM DTT) was added to samples, which were then sonicated in a water bath sonicator (Branson, Danbury CT) for 20 minutes at 25°C, incubated at 55°C for 10 minutes and incubated at 25°C for two hours. Octyl buffer (9.9M urea, 4% (w/v) octyl-β-D-glucopyranoside, 100 mM DTT) was added to samples and incubated at 25°C for one hour. Ampholytes (GE Healthcare, Piscataway NJ) and bromophenol blue were added to a final concentration of 0.5% (v/v) and 0.01% (v/v), respectively. Insoluble proteins were pelleted at 15,000×*g* for 30 minutes at 25°C. Soluble proteins were infiltrated into either 11 or 18 cm Immobiline DryStrip Gels (GE Healthcare) overnight and proteins were resolved as per manufacturer's instructions. The strips were then washed for 10 minutes in equilibration buffer (50 mM Tris pH 8, 6 M urea, 30% glycerol, 2% SDS) containing 1% DTT, followed by a second wash in equilibration buffer containing 1.25% iodoacetamide, and resolved by SDS-PAGE.

### ^35^S-labeling and immunoprecipitation

Parasites were metabolically labeled with [^35^S] methionine-cysteine (Perkin Elmer, Waltham MA) for 20 to 24 hours as previously described [43] and extracted in IP lysis buffer (1% (v/v) TX-100, 50 mM Tris-Cl pH 8, 150 mM NaCl, 5 mM EDTA) with 1∶100 protease inhibitors on ice for 10 minutes. Extracts were clarified at 13,000×*g* for 30 minutes at 4°C. Protein extracts were incubated with a 1∶1,000 dilution of anti-TgGAP45 [14] for 60 minutes at 4°C, followed by an additional 60 minute incubation with protein A or G conjugated to Sepharose (Zymed, San Francisco CA). The beads were washed with 60 volumes of IP lysis buffer. Proteins were eluted from the beads for 1D gel electrophoresis by boiling for five minutes in 1X SDS-PAGE sample buffer with or without reducing agent. Proteins were eluted for 2D gel electrophoresis (15 minutes at 25°C) in 1.2 mM Tris-Cl pH 7.4, 0.024% (w/v) SDS, 8 M urea, 3.2% (w/v) octyl-β-D-glucopyranoside, 80 mM DTT. Ampholytes and bromophenol blue were added to a final concentration of 0.5% (v/v) and 0.01% (v/v), respectively. Insoluble proteins were pelleted at 15,000×*g* for 30 minutes at 25°C.

### Effect of tachypleginA on isolated myosin motor complexes

^35^S-labeled myosin motor complexes were isolated by anti-TgGAP45 immunoprecipitation as described above. Protein A agarose beads with attached motor complexes were incubated in DMSO or 100 µM tachypleginA for 20 minutes at 25°C. Proteins were eluted from the beads with 1X SDS-PAGE sample buffer, resolved by SDS-PAGE and visualized by autoradiography.

### Cloning FLAG- and Myc-tagged TgMLC1

Total tachyzoite RNA was extracted using TRI reagent (Sigma) according to the manufacturer's instructions. The SuperScript first-strand synthesis system (Invitrogen) was used to generate oligo(dT)-primed first-strand cDNA from 5 µg RNA. The sequence of all primers used to create TgMLC1 constructs are listed in Table S1. Myc-tagged and FLAG-tagged TgMLC1 were amplified from total parasite cDNA using primer sets Myc-TgMLC1 5′/TgMLC1 3′ A or FLAG-TgMLC1 5′/TgMLC1 3′ A. TgMLC1 PCR products were cloned in the TOPO TA vector (Invitrogen) as per manufacturer's instructions. TgMLC1 was subsequently subcloned from the TOPO TA vector into the *T. gondii* expression vector, TUBIMC1YFP/sagCAT [44].

### Cloning TgMLC1 truncation mutants

To clone Myc-TgMLC1^1–193^ and Myc-TgMLC1^80–213^ expression constructs, the Myc-TgMLC1 TOPO plasmid was used as a template for PCR with primers Myc-TgMLC1 5′/TgMLC1 aa 193 3′ and Myc-TgMLC1 aa 80 5′/TgMLC1 3′ A, respectively. PCR products were cloned in the TOPO TA vector and subcloned using BglII and AvrII restriction sites into TUBIMC1YFP/sagCAT. To clone TgMLC1^1–79^YFP, TgMLC1^40–79^YFP and TgMLC1^60–79^YFP, Myc-TgMLC1 TOPO was used as a template to amplify aa1–79, aa40–79 and aa60–79 of TgMLC1 using primers TgMLC1 5′ B/TgMLC1 79 3′, TgMLC1aa40 5′/TgMLC1 79 3′ and TgMLC1 aa60 5′/TgMLC1 79 3′, respectively. PCR products were cloned in the TOPO TA vector and subcloned using BglII and AvrII restriction sites into TUBIMC1YFP/sagCAT.

### Immunofluoresence microscopy

Intracellular parasites were grown on confluent HFF monolayers for approximately 24 hours. Cells were fixed with 2% (v/v) formaldehyde diluted in PBS for 15 minutes at 25°C. Cells were permeabilized with 0.25% (v/v) TX-100 diluted in PBS for 10 minutes at 25°C. Primary and secondary antibodies were diluted in PBS containing 0.5% (w/v) BSA, filtered through a 0.22 µm filter and incubated with cells for 15 minutes at 25°C. Anti-IMC1 and anti-GFP antibodies were diluted 1∶1,000. Secondary antibodies conjugated to either Alexa 488 or 546 (Invitrogen) were diluted 1∶500.

### Myosin motor complex isolation for *in vitro* motility assays

5−8×10^9^ extracellular parasites were treated with DMSO or 100 µM tachypleginA and extracted in 3 ml FLAG lysis buffer (10 mM imidazole pH 7, 300 mM NaCl, 1 mM EGTA, 5 mM MgCl_2_, 1% (v/v) TX-100, 2 mM ATP, 2 mM DTT and 1∶100 protease inhibitor cocktail) on ice for 10 minutes. Protein extracts were clarified at 10,000×*g* for 30 minutes. Before use, FLAG affinity resin (Sigma) was equilibrated in 0.1M glycine pH 3.5 and washed with FLAG lysis buffer as per manufacturer's instructions. Protein extracts and approximately 400 µl FLAG affinity resin were agitated for two hours at 4°C. Resins were extensively washed with FLAG wash buffer (10 mM imidazole pH 7, 300 mM NaCl, 1 mM EDTA, 1 mM ATP, 1 mM DTT, 1% (v/v) TX-100 and 1∶500 protease inhibitors) to remove any unbound proteins, and FLAG-TgMLC1 and its associated proteins were eluted from the resin using 100 µl 0.2 mg/ml FLAG peptide (Sigma) in FLAG wash buffer, agitating for 10 minutes. Remaining protein bound to the beads was eluted with a second wash of 50 µl of 0.2 mg/ml FLAG peptide. Eluates were combined and either stored on ice or diluted with 1/3 volume 100% (v/v) glycerol and stored at -20°C and used for functional assays within 48 hours. Isolated myosin motor complexes were resolved by SDS-PAGE along with protein standards (Phosphorylase b 94kDa; Albumin 67kDa; Ovalbumin 43kDa; Carbonic anhydrase 30kDa; Trypsin inhibitor 20.1kDa and α-lactalbumin 14.4kDa (Pharmacia LKB, Piscataway, NJ)) of known concentration. Gels were stained with SYPRO Ruby (Bio-Rad) as per manufacturer's instructions and imaged using a Bio-Rad FX-imager. The fluorescence intensity of the protein standards was compared to the fluorescence intensity of TgMyoA to determine the amount of myosin isolated from both DMSO- and tachypleginA-treated parasites.

### TgMyoA *in vitro* motility assays

Isolated myosin motor complexes were added to flow cells [33] and incubated for two minutes to allow protein to adhere to nitrocellulose-coated coverslips, followed by the addition of 0.5 mg/ml BSA in Actin Buffer (25 mM KCl, 25 mM imidazole, 1 mM EGTA, 4 mM MgCl_2_, 10 mM DTT, pH 7.4) to block any unoccupied sites. The flow cells were then washed with Actin Buffer three times, twice infused with TRITC-phalloidin labeled chicken skeletal muscle actin filaments [33] in Actin Buffer containing an oxygen scavenger system (0.1 mg/ml glucose oxidase, 0.018 mg/ml catalase, 2.3 mg/ml glucose), and incubated for 30 seconds. Finally, flow cells were washed with Actin Buffer containing 0.5% (v/v) methylcellulose and 1 mM ATP. Myosin activity was monitored by video microscopy and actin filament velocities were determined as previously described [31],[33]. For each myosin concentration at least 19 filaments (mean 42, range 19–82) from five different areas of the coverslip were tracked at one frame per second until the filament exited the field or stopped moving for two or more frames. Stationary filaments as well as those that were undergoing reptation (*i.e.*, exhibiting random motion parallel to the filament's long axis) were excluded from the analysis.

To determine the TgMyoA duty ratio, the amount of myosin capable of interacting with a given length of actin filament was determined based on the concentration of TgMyoA added to the coverslip and surface area of the coverslip as previously described [33]. Actin filament lengths were measured as previously described [31], thus allowing the number of myosin heads capable of interacting with any given actin filament to be estimated. Duty ratios were determined by fitting data to the equation V =  (*a*×V_max_)×[1−(1−f)^n^] where V is the measured actin filament velocity, f is duty ratio, n is the number of myosin heads capable of interacting with the actin filament, and *a* is an “efficiency factor,” which is a parameter of the fit [33],[45]. Data from a total of 195 (control) and 235 (tachypleginA) filaments were used to generate the fits shown in Fig. 5C. The absolute duty ratios obtained from the fits assume that all heads on the motility surface are functional. This may not be the case if the number of functional heads is less such that the duty ratios are underestimates. Since there is no *a priori* reason to assume that the DMSO- and tachypleginA-treated preparations would bind differentially to the motility surface, any differences in the duty ratios between the control and compound-treated samples are assumed to reflect true relative differences. Data were analyzed using GraphPad Prism v.5.01 software.

### Isolation of TgMLC1 for mass spectrometry analysis

For SILAC experiments cells were grown essentially as described [28]. Briefly, infected HFF cells were cultured at 37°C and 5% CO_2_ for four days in DMEM deficient in L-arginine and L-lysine (prepared by Cambridge Isotope Laboratories, Inc. Andover, MA) supplemented with 10% dialyzed fetal bovine serum (HyClone, Logan, UT); 50 units/ml penicillin and 50 µg/ml streptomycin (Invitrogen, Carlsbad, CA); and either 146 mg/l unlabeled L-lysine and 84 mg/l unlabeled L-arginine (“light”), or 146 mg/l ^13^C_6_-, ^15^N_2_-L-lysine and 84 mg/l ^13^C_6_-, ^15^N_4_-L-arginine (“heavy”, Cambridge Isotope Laboratories, Inc., Andover, MA). Both heavy and light media were also supplemented with 30–40 mg/l of unlabeled L-proline. Heavy parasites were treated with tachypleginA and light parasites were treated with DMSO for 20 minutes at 25°C. Parasites were extracted in FLAG lysis buffer and myosin motor complexes were purified using FLAG affinity resin as described above. Heavy and light motor complexes were combined and precipitated with 10 volumes of acetone at −20°C overnight. Proteins were pelleted at 10,000×*g* for 30 minutes at 4°C, incubated in 90% acetone at −20°C for 30 minutes and pelleted as before. Pellets were allowed to dry at 25°C and then solubilized for 2D gel electrophoresis as described above. Gels were stained with Colloidal Coomassie Blue and spots were excised and subjected to in-gel tryptic digestion as described previously [46]. Mass measurements were made in an LTQ-Orbitrap hybrid mass spectrometer (Thermo Fisher Scientific, San Jose, CA) which was set up with a liquid chromatography interface essentially as described [47]. Analysis of samples employed a precursor MS1 scan in the Orbitrap at 30,000 resolution, followed by 10 MS/MS scans in the LTQ linear ion trap on the most abundant ions identified in the precursor scan. Dynamic exclusion was set at 30 seconds with a repeat count of 3. Initial Sequest analysis of tandem mass spectra was conducted using the TgMLC1 amino acid sequence, requiring no enzyme specificity, allowing a 30ppm window around the precursor mass, and allowing differential mass additions of 15.99491 for methionine, 10.00827 for arginine and 8.01420 for lysine. A static increase of 57.02146 was set on cysteines for carbamidomethylation. Quantification of SILAC data was done manually as described [48]. For the non-SILAC sample analysis Sequest searches of tandem mass spectra obtained in the LTQ linear ion trap were conducted with a 2Da mass tolerance of the precursor and differential modification of cysteine from 1–353 (*i.e.*, 353 searches were performed, with the differential modification of cysteine increasing incrementally by one Da each search). Differential searches were also performed for potential phosphorylation, acetylation and mono-, di-, and tri-methylation of peptide V_46_GEYDGACESPSCR_59_.

## Supporting Information

Figure S1 Identification of two analogs of tachypleginA that induce the TgMLC1 electrophoretic mobility shift. Structures of tachypleginA, A-2 and A-3 (left) and demonstration by anti-TgMLC1 western blot (right) that all three compounds induce a similar electrophoretic mobility shift of TgMLC1 at 100 µM. Unmodified and modified forms of TgMLC1 are indicated by blue and red arrowheads, respectively. All three compounds inhibit parasite motility and invasion at 100 µM (data not shown).
(0.13 MB TIF)

Figure S2 Myc-tagged TgMLC1 undergoes the electrophoretic mobility shift in response to tachypleginA treatment. Myc-TgMLC1-WT expressing parasites were treated with 100 µM tachypleginA or an equivalent amount of DMSO, extracted, resolved by 2D gel electrophoresis and analyzed by western blot. Myc-TgMLC1-WT (green brackets) and endogenous TgMLC1 (blue brackets) are both modified by tachypleginA.
(0.33 MB TIF)

Figure S3 Localization of TgMLC1 truncation mutants. Dual immunofluorescence labeling of parasites expressing Myc-TgMLC1-WT, Myc-TgMLC1^80–213^ and Myc-TgMLC1^1–193^ with antibodies against TgGAP45 (red) and the Myc epitope tag (green). Myc-TgMLC1-WT and Myc-TgMLC1^1–193^ localize to the parasite periphery, whereas Myc-TgMLC1^80–213^ localizes to the cytosol.
(0.51 MB TIF)

Figure S4 MS/MS spectrum of the peptide V_46_GEYDGACESPSCR_59_. Low energy collision-induced dissociation MS/MS spectrum for the doubly-charged ion of the V_46_GEYDGACESPSCR_59_ peptide. This spectrum was observed multiple times in both heavy and light forms during the same chromatographic time that quantitative mass spectrometry (SILAC) measurements were taken on the precursor ions (see Fig. 4). C# indicates a carbamidomethyl cysteine residue generated by alkylation with iodoacetamide prior to running the 2D gels.
(0.75 MB TIF)

Figure S5 Estimation of the relative abundance of peptide V_46_GEYDGACESPSCR_59_ in the two forms of TgMLC1. Using Xcalibur software (Thermo Scientific), the relative abundance of V_46_GEYDGACESPSCR_59_ in the upper and lower forms of TgMLC1 was semi-quantitatively compared to the relative abundance of a dominant background ion, [(Si(CH_3_)_2_O)_6_ + H^+^]^+^ (monoisotopic mass = 445.12). The relative abundance of V_46_GEYDGACESPSCR_59_ to the background ion in the upper spot was approximately 225 times greater than that in the lower spot (*i.e.*, the monoisotopic peak of the light V_46_GEYDGACESPSCR_59_ peptide in the lower spot had to be raised 225 times in value to achieve the same signal observed in the upper spot). C# indicates a carbamidomethyl cysteine residue generated by alkylation with iodoacetamide prior to running the 2D gels.
(0.39 MB TIF)

Figure S6 Determination of the TgMyoA concentration in purified motor complex preparations. The concentration of TgMyoA recovered from FLAG-TgMLC1 expressing parasites treated with either DMSO or 100 µM tachypleginA was determined by staining the SDS-PAGE-resolved preparations with SYPRO Ruby and comparing the fluorescence intensity of the TgMyoA band with known amounts of four protein standards (see Materials and Methods). Numbers on the left indicate molecular mass in kDa and numbers above lanes 1–7 indicate the amount of each standard (in µg) loaded in that lane.
(0.40 MB TIF)

Text S1 Supplemental methods and references
(0.30 MB PDF)

Table S1 TgMLC1 primer sequences.
(0.05 MB PDF)

Video S1 *In vitro* motility of purified TgMyoA motor complexes from tachypleginA-treated parasites. FLAG-TgMLC1-expressing parasites were treated for 15 minutes at 24°C with 100 µM tachypleginA (final DMSO concentration, 0.25% (vol:vol)). Myosin motor complexes were isolated from the treated parasites by anti-FLAG affinity chromatography and adsorbed to nitrocellulose-coated coverslips at a TgMyoA concentration of 8.6 µg/ml. TRITC-phalloidin-labeled actin filaments were added to the immobilized motor complexes, and actin filament displacement was captured by videomicroscopy as described in Materials and Methods. Filament displacement is shown in real time; scale bars = 5 µm.
(7.8 MB ZIP)

Video S2 *In vitro* motility of purified TgMyoA motor complexes from control parasites. FLAG-TgMLC1-expressing parasites were treated for 15 minutes at 24°C with an amount of DMSO equivalent to that used in Video S1 (0.25% (vol:vol)). Myosin motor complexes were isolated from the treated parasites by anti-FLAG affinity chromatography and adsorbed to nitrocellulose-coated coverslips at a TgMyoA concentration of 7.4 µg/ml. TRITC-phalloidin-labeled actin filaments were added to the immobilized motor complexes, and actin filament displacement was captured by videomicroscopy as described in Materials and Methods. Filament displacement is shown in real time; scale bars = 5 µm.
(7.7 MB ZIP)
