# Supplementary figures and images for: A Small-Molecule Inhibitor of T. gondii Motility Induces the Posttranslational Modification of Myosin Light Chain-1 and Inhibits Myosin Motor Activity
Source: PLoS Pathog. 2010 Jan 15;6(1):e1000720. doi: 10.1371/journal.ppat.1000720 (PMC2800044; doi:10.1371/journal.ppat.1000720)

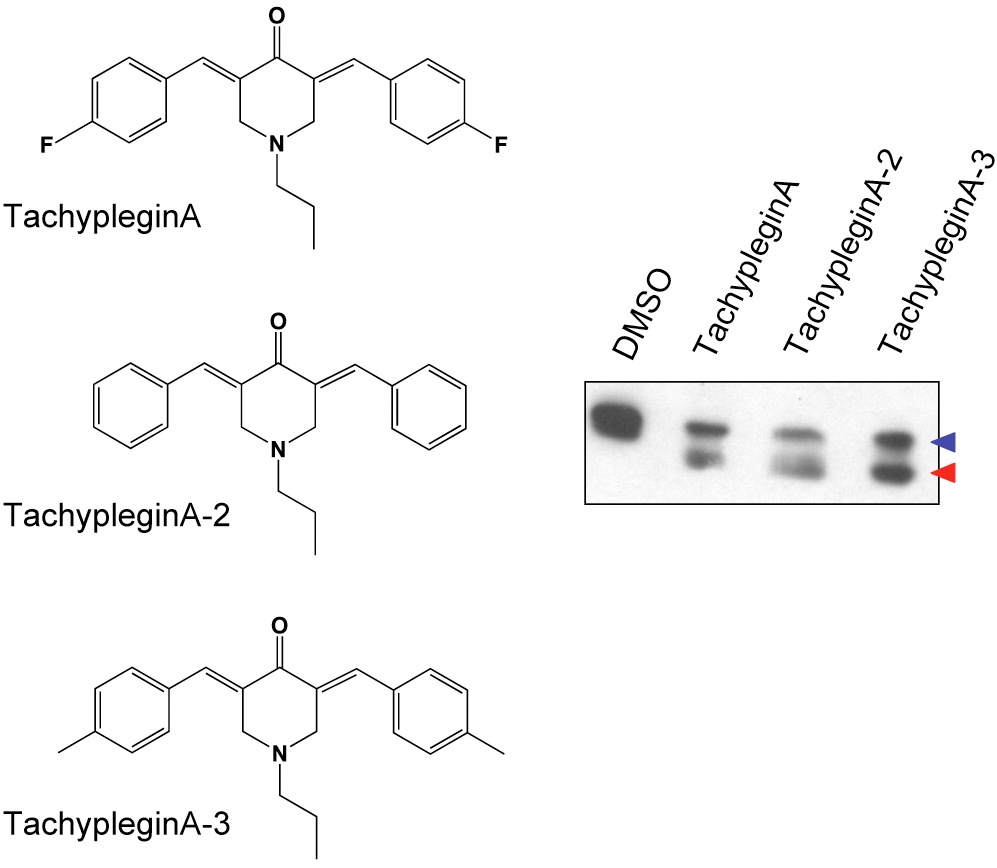

Supplement: Figure S1 — Identification of two analogs of tachypleginA that induce the TgMLC1 electrophoretic mobility shift. Structures of tachypleginA, A-2 and A-3 (left) and demonstration by anti-TgMLC1 western blot (right) that all three compounds induce a similar electrophoretic mobility shift of TgMLC1 at 100 µM. Unmodified and modified forms of TgMLC1 are indicated by blue and red arrowheads, respectively. All three compounds inhibit parasite motility and invasion at 100 µM (data not shown). (0.13 MB TIF) [file ppat.1000720.s001.tif]

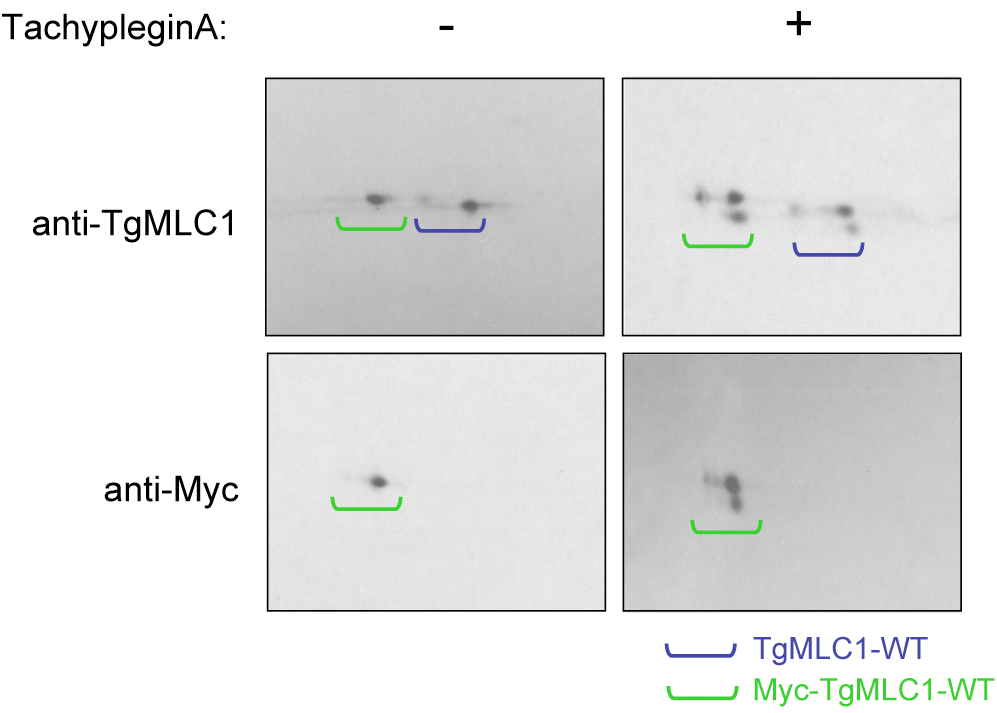

Supplement: Figure S2 — Myc-tagged TgMLC1 undergoes the electrophoretic mobility shift in response to tachypleginA treatment. Myc-TgMLC1-WT expressing parasites were treated with 100 µM tachypleginA or an equivalent amount of DMSO, extracted, resolved by 2D gel electrophoresis and analyzed by western blot. Myc-TgMLC1-WT (green brackets) and endogenous TgMLC1 (blue brackets) are both modified by tachypleginA. (0.33 MB TIF) [file ppat.1000720.s002.tif]

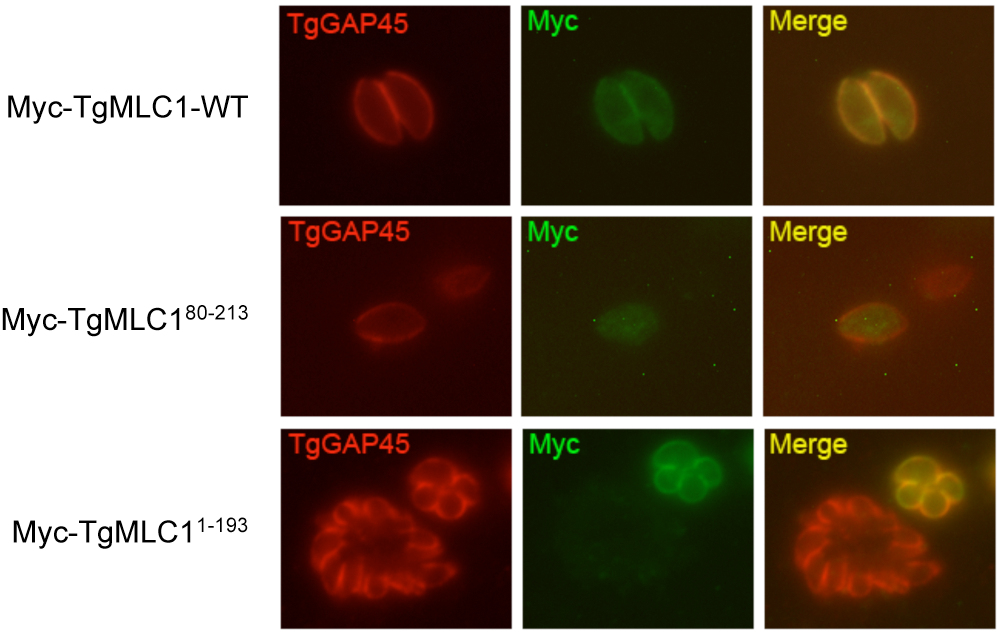

Supplement: Figure S3 — Localization of TgMLC1 truncation mutants. Dual immunofluorescence labeling of parasites expressing Myc-TgMLC1-WT, Myc-TgMLC180–213 and Myc-TgMLC11–193 with antibodies against TgGAP45 (red) and the Myc epitope tag (green). Myc-TgMLC1-WT and Myc-TgMLC11–193 localize to the parasite periphery, whereas Myc-TgMLC180–213 localizes to the cytosol. (0.51 MB TIF) [file ppat.1000720.s003.tif]

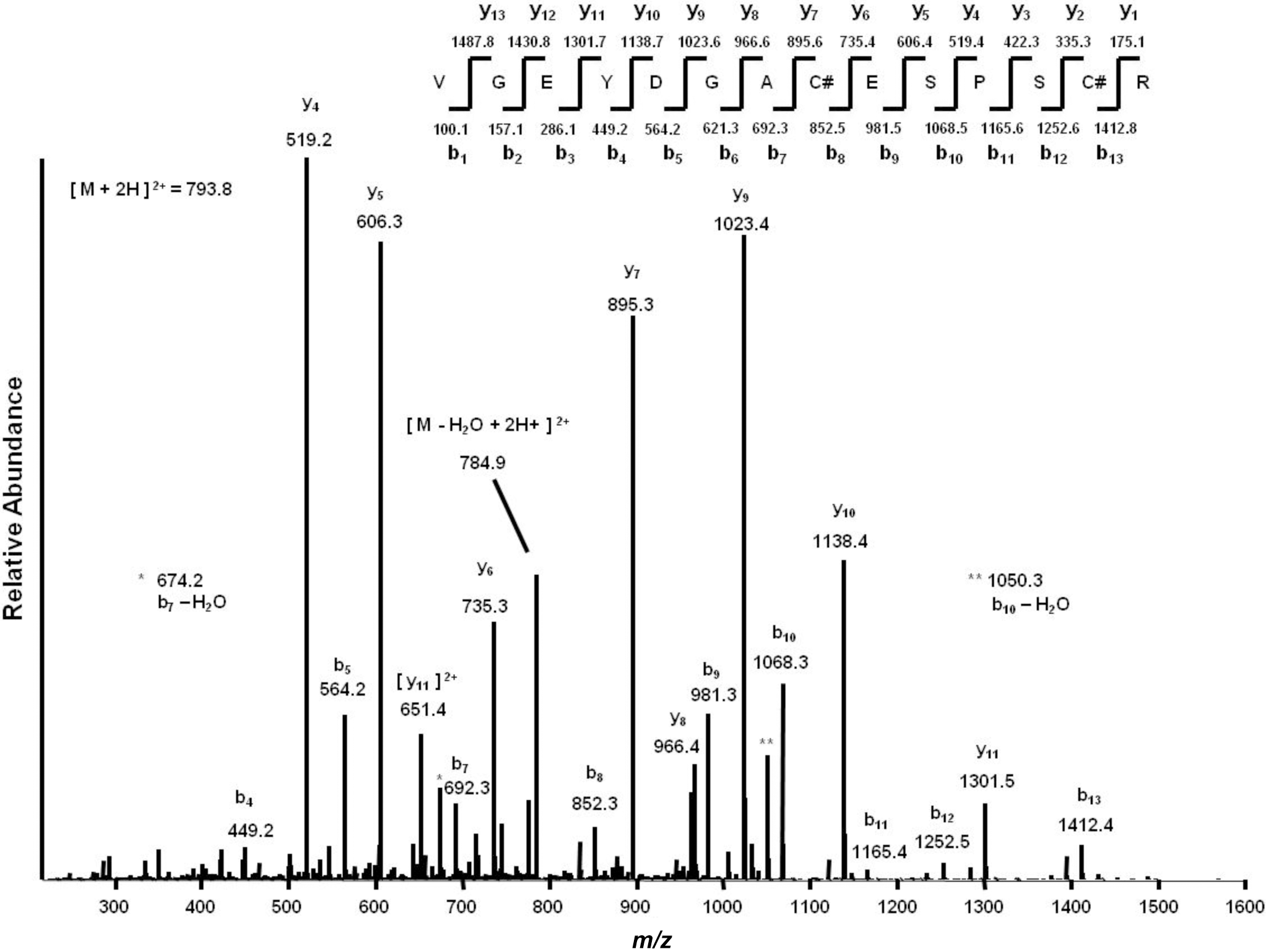

Supplement: Figure S4 — MS/MS spectrum of the peptide V46GEYDGACESPSCR59. Low energy collision-induced dissociation MS/MS spectrum for the doubly-charged ion of the V46GEYDGACESPSCR59 peptide. This spectrum was observed multiple times in both heavy and light forms during the same chromatographic time that quantitative mass spectrometry (SILAC) measurements were taken on the precursor ions (see Fig. 4). C# indicates a carbamidomethyl cysteine residue generated by alkylation with iodoacetamide prior to running the 2D gels. (0.75 MB TIF) [file ppat.1000720.s004.tif]

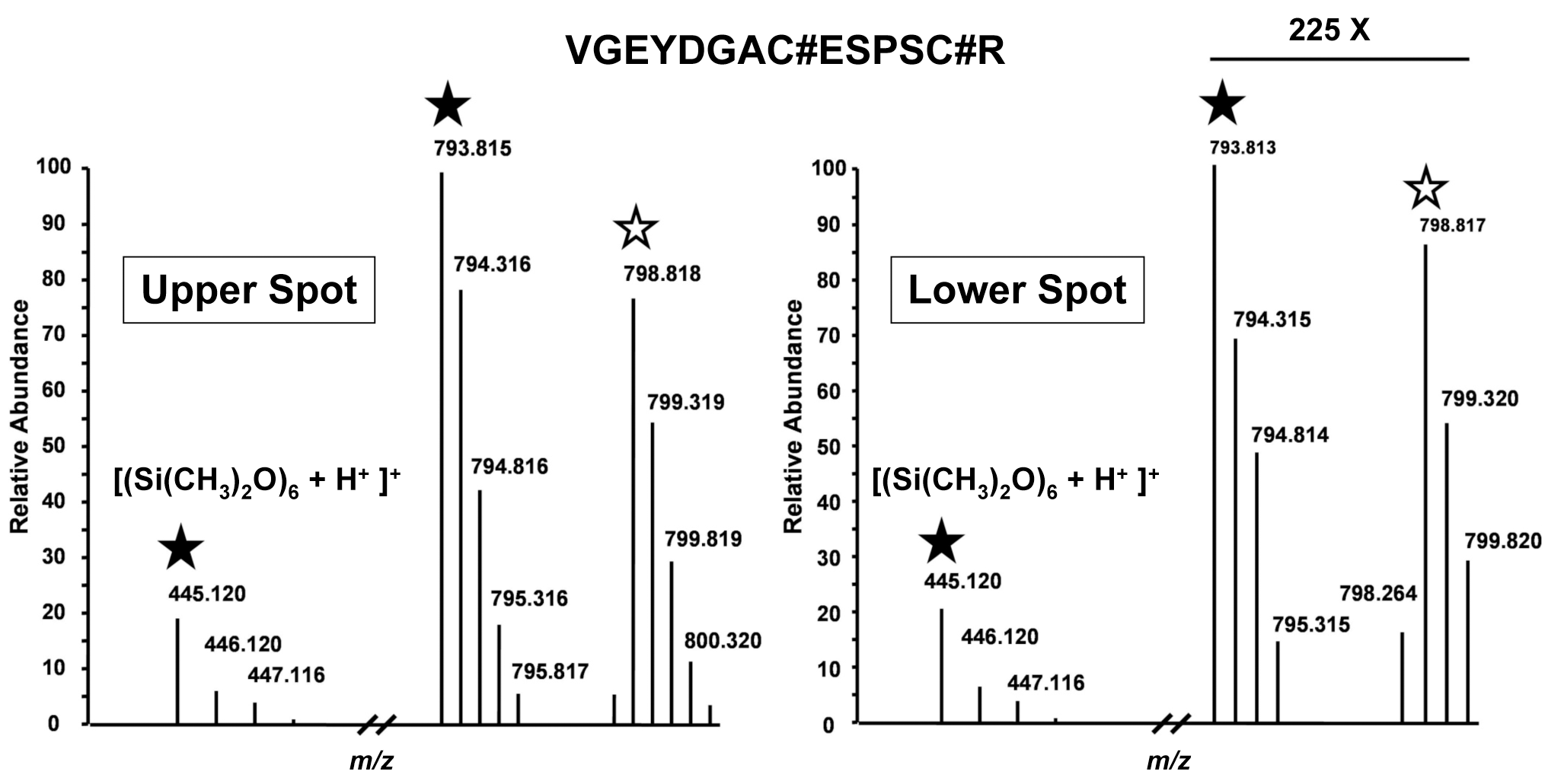

Supplement: Figure S5 — Estimation of the relative abundance of peptide V46GEYDGACESPSCR59 in the two forms of TgMLC1. Using Xcalibur software (Thermo Scientific), the relative abundance of V46GEYDGACESPSCR59 in the upper and lower forms of TgMLC1 was semi-quantitatively compared to the relative abundance of a dominant background ion, [(Si(CH3)2O)6 + H+]+ (monoisotopic mass = 445.12). The relative abundance of V46GEYDGACESPSCR59 to the background ion in the upper spot was approximately 225 times greater than that in the lower spot (i.e., the monoisotopic peak of the light V46GEYDGACESPSCR59 peptide in the lower spot had to be raised 225 times in value to achieve the same signal observed in the upper spot). C# indicates a carbamidomethyl cysteine residue generated by alkylation with iodoacetamide prior to running the 2D gels. (0.39 MB TIF) [file ppat.1000720.s005.tif]

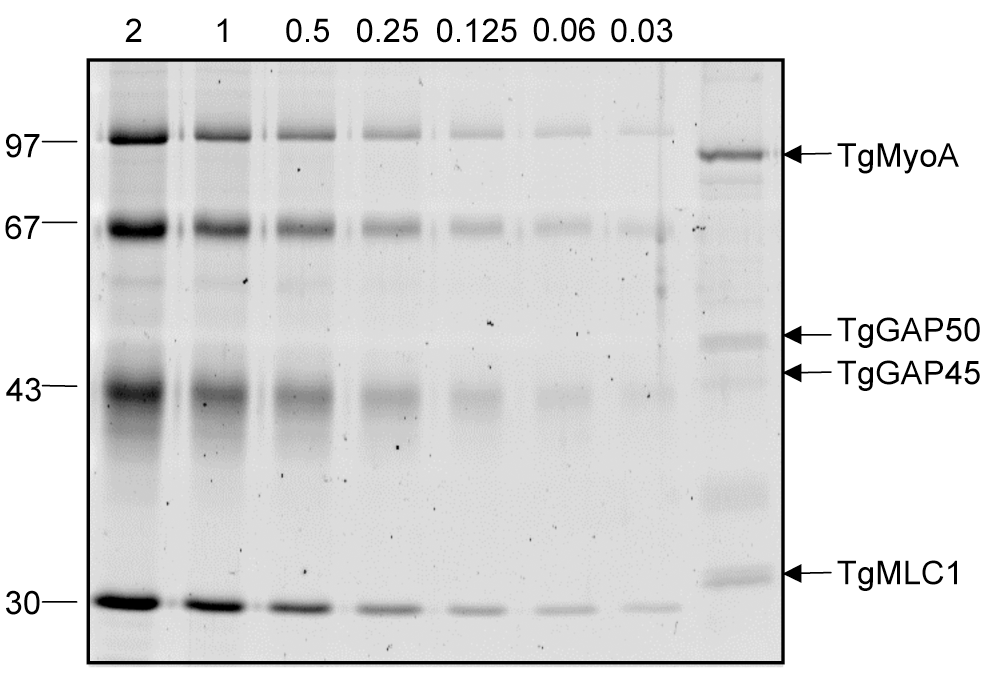

Supplement: Figure S6 — Determination of the TgMyoA concentration in purified motor complex preparations. The concentration of TgMyoA recovered from FLAG-TgMLC1 expressing parasites treated with either DMSO or 100 µM tachypleginA was determined by staining the SDS-PAGE-resolved preparations with SYPRO Ruby and comparing the fluorescence intensity of the TgMyoA band with known amounts of four protein standards (see Materials and Methods). Numbers on the left indicate molecular mass in kDa and numbers above lanes 1–7 indicate the amount of each standard (in µg) loaded in that lane. (0.40 MB TIF) [file ppat.1000720.s006.tif]
