# Supplementary material for: A Small-Molecule Inhibitor of T. gondii Motility Induces the Posttranslational Modification of Myosin Light Chain-1 and Inhibits Myosin Motor Activity
Source: PLoS Pathog. 2010 Jan 15;6(1):e1000720. doi: 10.1371/journal.ppat.1000720 (PMC2800044; doi:10.1371/journal.ppat.1000720)
Supplement: Text S1 — Supplemental methods and references (0.30 MB PDF) [file ppat.1000720.s007.pdf]

**SUPPLEMENTAL MATERIAL**

**Supplemental methods**

**Chemistry experimental:**

Previous reports on the synthesis of arylidene ketones of this general type have been reported by us [1] and others [2]. The synthesis of two active analogs of tachypleginA using this approach is described below. To the best of our knowledge, this is the first time a parallel synthesis protocol for the rapid and robust generation of structures of this type has been reported.

**General procedure for the synthesis of tachypleginA and its analogs:** In each test tube of a Buchi syncore parallel reactor a different aldehyde (4.1 mmol) was added to a suspension of differently substituted cyclic ketones (2.0 mmol) in acetic acid (5.0 ml) saturated with dry hydrogen chloride gas. The reactions were stirred at room temperature for 24 h and then saturated aqueous potassium carbonate solution (10.0 ml) and acetone (4.0 ml) were added. The resultant mixtures were stirred for 0.5 h after which a precipitate of the desired compound formed in each tube and was collected by filtration using automated filtration equipment. After washing each precipitate with MeOH (3 x 10 ml), the solid was dissolved in warm 98% ethanol and allowed to crystallize. After crystallization, the compounds were analysed by $^1$H-NMR spectroscopy and LC-MS to confirm the structure and purity.

**TachypleginA** (*N*-Propyl-3,5-bis-*(E)*-[1-*p*-fluoromethylidene]piperidin-4-one)

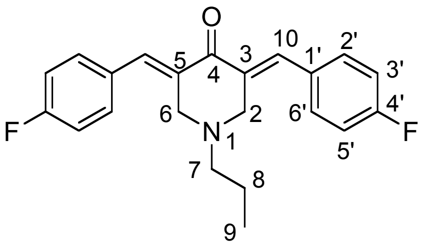


Yield 82%; *m.p.*: 121-122 °C; *$^1$H-NMR (300 MHz, $CDCl_3$)*: δ 7.94 (s, 2H, $\underline{H}_{10}$), 7.43-7.36 (m, 4H, $\underline{H}_{3',5'}$), 7.19-7.11 (m, 4H, $\underline{H}_{2',6'}$), 4.17 (s, 4H, $\underline{H}_{2,6}$), 2.69 (t, 2H, $^3J$= 7.7, $\underline{H}_7$), 1.59-1.48 (m, 2H, $\underline{H}_8$), 0.87 (t, 3H, $^3J$= 7.4, $\underline{H}_9$); *LRMS* (ES$^+$) *(MeOH)*: *m/z* 354.18 $[M+H]^+$ (100%).

**TachypleginA-2** (*N*-Propyl-3,5-bis-(1-phenylmethylidene)piperidin-4-one):

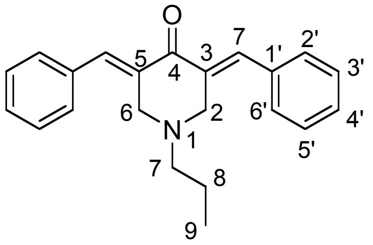


Yield 78%; ***m.p.***: 114.0-114.5 °C; $^{1}$***H-NMR*** *(300 MHz, $CDCl_3$)*: δ 7.75 (s, 2H, $H_{10,}$), 7.40-7.26 (m, 10H, $H_{2'-6'}$), 3.76 (s, 4H, $H_{2,6}$), 2.43 (t, 2H, $^{3}J$= 7.7, $H_7$), 1.37 (m, 2H, $^{3}J$= 7.7, 7.4, $H_8$), 0.80 (t, 3H, $^{3}J$= 7.4, $H_9$);$^{13}$***C-NMR*** *(75.5 MHz, $CDCl_3$)*: δ 187.9 ($C_4$), 136.8 ($C_{10}$), 135.7 ($C_{3,5}$), 133.8 ($C_{1'}$), 130.8 ($C_{2'}$), 129.4 ($C_{4'}$), 129.0 ($C_{3'}$), 59.7 ($C_7$), 55.2 ($C_{2,6}$), 20.8 ($C_8$), 12.2 ($C_9$); ***IR*** *(KBr)* $\nu_{max}$: 2959 (m), 2748 (m), 1669.3 (m) (C=O), 1610 (s) (C=C-Ph) $cm^{-1}$; ***LRMS*** **($ES^+$)** *(MeOH)*: *m/z* 318.12 $[M+H]^+$ (100%); ***HRMS*** **($ES^+$)** *(MeOH)*: *m/z* calc'd for $C_{22}H_{24}NO$: 318.1858, found: 318.1851 (-2.3 ppm); Anal. calc'd for $C_{22}H_{23}NO$: 83.24%C 7.30%H 4.41%N, found: 83.24%C 7.50%H 4.34% N. Further recrystallization from 98% ethanol gave yellow needle-like crystals of sufficient quality for small molecule X-ray crystallographic analysis.

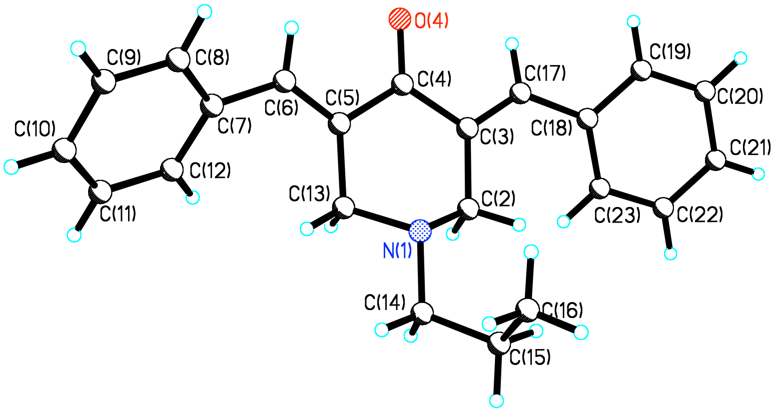


| CRYSTAL DATA AND STRUCTURE REFINEMENT OF **TachypleginA-2** | | |
|---|---|---|
| Empirical formula | C22 H23 N O | |
| Formula weight | 317.41 | |
| Temperature | 93(2) K | |
| Wavelength | 0.71073 Å | |
| Crystal system | Orthorhombic | |
| Space group | P2(1)2(1)2(1) | |
| Unit cell dimensions | a = 6.0725(10) Å | α= 90°. |
| | b = 12.679(2) Å | β= 90°. |
| | c = 23.050(4) Å | γ = 90°. |
| Volume | 1774.7(5) $Å^3$ | |
| Z | 4 | |
| Density (calculated) | 1.188 $Mg/m^3$ | |
| Absorption coefficient | 0.072 $mm^{-1}$ | |
| F(000) | 680 | |

| | | |
|---|---|---|
| Crystal size | 0.300 x 0.100 x 0.050 mm$^3$ | |
| Theta range for data collection | 3.10 to 25.34°. | |
| Index ranges | 0<=h<=7, 0<=k<=14, -26<=l<=26 | |
| Reflections collected | 3041 | |
| Independent reflections | 3041 [R(int) = 0.0000] | |
| Completeness to theta = 25.34° | 94.0 % | |
| Absorption correction | MULTISCAN | |
| Max. and min. transmission | 1.0000 and 0.7355 | |
| Refinement method | Full-matrix least-squares on F$^2$ | |
| Data / restraints / parameters | 3041 / 0 / 218 | |
| Goodness-of-fit on F$^2$ | 1.052 | |
| Final R indices [I>2sigma(I)] | R1 = 0.0373, wR2 = 0.0775 | |
| R indices (all data) | R1 = 0.0444, wR2 = 0.0803 | |
| Absolute structure parameter | 1.8(18) | |
| Extinction coefficient | 0.0119(11) | |
| Largest diff. peak and hole | 0.155 and -0.154 e.Å$^{-3}$ | |

**Tachyplegin A-3 (***N*-Propyl-3,5-bis-*(E)*-[1-*p*-tolylmethylidene]piperidin-4-one)**:**

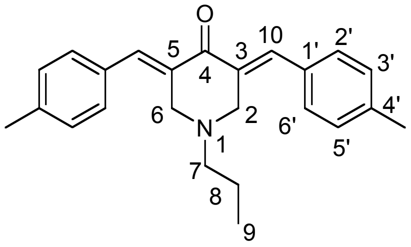


Isolated as an orange crystalline solid; Yield 45%; ***$^1$H-NMR** (300 MHz, $CDCl_3$)*: 7.79 (br s, 2H, $\underline{H}_{10}$), 7.31 (d (AB system), 4H, $^3J$ = 8.0, $\underline{H}_{2',6'}$), 7.23 (d (AB system), 4H, $^3J$ = 8.0, $\underline{H}_{3',5'}$), 3.83 (d, 4H, $^4J$ = 1.8, $\underline{H}_{2,6}$), 2.50 (dd, 2H, $^3J$ = 7.4, 5.8, $\underline{H}_7$), 2.39 (s, 6H, C$\underline{H}_3$), 1.53-1.39 (m, 2H, $\underline{H}_8$), 0.85 (t, 3H, $^3J$ = 7.4, $\underline{H}_9$); ***$^{13}$C-NMR** (75.5 MHz, $CDCl_3$)*: δ 188.1 ($\underline{C}_4$), 139.7 ($\underline{C}_{4'}$), 136.8 ($\underline{C}_{10}$), 133.0 ($\underline{C}_{3,5}$), 132.9 ($\underline{C}_{1'}$), 130.9 ($\underline{C}_{3',5'}$), 129.7 ($\underline{C}_{2',6'}$), 59.7 ($\underline{C}_7$), 55.2 ($\underline{C}_{2,6}$), 21.9 ($\underline{C}H_3$), 20.9 ($\underline{C}_8$), 12.2 ($\underline{C}_9$).***LRMS*** **(ES$^+$)** *(MeOH)*: *m/z* 346 $[M+H]^+$ (90), 368 $[M+Na]^+$ (10%).

**Supplemental References.**

1. Catti F, Kiuru PS, Slawin AMZ, Westwood N (2008) The synthesis of highly functionalized pyridines using Ghosez-type reactions of dihydropyrazoles. Tetrahedron 63: 9561-9566.

2. Dimmock JR, Padmanilayam MP, Puthucode RN, Nazarali AJ, Motaganahalli NL, Zello GA, Quail JW, Oloo EO, Kraatz HB, Prisciak JS, Allen TM, Santos CL, Balzarini J, De Clercq E, Manavathu EK (2001) A conformational and structure-activity relationship study of cytotoxic 3,5-bis(arylidene)-4-piperidones and related N-acryloyl analogues. J Med Chem 44: 586-593.
