# Supplementary material for: A Small-Molecule Inhibitor of T. gondii Motility Induces the Posttranslational Modification of Myosin Light Chain-1 and Inhibits Myosin Motor Activity
Source: PLoS Pathog. 2010 Jan 15;6(1):e1000720. doi: 10.1371/journal.ppat.1000720 (PMC2800044; doi:10.1371/journal.ppat.1000720)
Supplement: Table S1 — TgMLC1 primer sequences. (0.05 MB PDF) [file ppat.1000720.s008.pdf]

Supplementary Table 1. TgMLC1 primer sequences.

	Name	Sequence	Target
1	TgMLC1 5' B	GGAAGATCTATGAGCAAGGTCGAGAAG	5' coding region of TgMLC1 with BglII site
2	TgMLC1 3' A	CCTCCTAGGTTACTCCCTTCGCTCG	3' coding region of TgMLC1 with AvrII site
3	Myc-TgMLC1 5'	GGAAGATCTATGGAGCAGAAGCTCATCTC CGAGGAGGACCTGAGCAAGGTCGAGAAG	5' coding region of TgMLC1 with 5' Myc tag coding sequence
4	FLAG-TgMLC1 5'	AGATCTATGGATTACAAGGATGACGACGAT AAGATGAGCAAGGTCGAGAA	5' coding region of TgMLC1 with 5' FLAG tag coding sequence
5	TgMLC1 79 3'	CCTCCTAGGCTCCACCATCTCCTCGAGAG	TgMLC1 coding region binding from codon 79
6	TgMLC1 UTR 5'	AACGAAATTGTCCAGCCTT	Binds 28bp upstream of TgMLC1 ATG start in 5' UTR
7	TgMLC1 UTR 3'	AAACAGCTGTCCACACACAAA	Binds 34bp downstream of TgMLC1 stop codon in 3' UTR
8	Myc-TgMLC1 aa 80 5'	GGAAGATCTATGGAGCAGAAGCTCATCTCC GAGGAGGACCTGGCCGACGAAATGTATGCG CG	Binds to TgMLC1 coding region starting at codon 80 with 5' Myc coding sequence
9	TgMLC1 aa 193 3'	CCTCCTAGGTTAGAAGTACTCCGCAGCCAA	Binds to TgMLC1 coding region starting at codon 193
10	TgMLC1 aa 40 5'	AGATCTATGCGCCCGGAACCGAAGGTGGGG GAGTA	Binding to TgMLC1 coding region starting at codon 40
11	TgMLC1 aa 60 5'	AGATCTATGGAGGGGGGCGCCCTGCGGCA GACGAA	Binding to TgMLC1 coding region starting at codon 60
